# Supplementary material for: Diet Quality and Mortality among Chinese Adults: Findings from the China Health and Nutrition Survey
Source: Nutrients. 2023 Dec 27;16(1):94. doi: 10.3390/nu16010094 (PMC10780502; doi:10.3390/nu16010094)
Supplement: Supplementary file 1 [file nutrients-16-00094-s001.zip › Supplemental Figures.pdf]

Subgroups	Number of deaths / total sample size	Multivariable-adjusted HR (95%CI)	P-interaction
Age group*			0.76
<47	37/6323	0.99 (0.95, 1.04)	
≥47	424/6130	0.99 (0.98, 1.01)	
Sex			0.22
Female	265/5894	0.99 (0.98, 1.01)	
Male	196/6559	1.01 (0.99, 1.03)	
Baseline year			0.06
2004	413/6992	1.01 (0.99, 1.02)	
2006 or 2009 or 2011	48/5461	0.98 (0.95, 1.02)	
Educational level			0.21
Primary school and lower	340/4317	1.00 (0.99, 1.02)	
Middle school, college and higher	118/8066	0.99 (0.96, 1.01)	
Marital status			0.07
Married	350/10441	0.99 (0.98, 1.01)	
Never married, separated, divorced, or widowed	110/1901	1.01 (0.99, 1.05)	
Household income per year			0.77
Low	229/3993	1.00 (0.98, 1.03)	
Medium	157/4196	0.99 (0.97, 1.01)	
High	73/4150	1.00 (0.97, 1.03)	
Self-reported health status			0.46
Good and above	151/4980	1.01 (0.98, 1.03)	
Fair	195/2600	1.01 (0.99, 1.03)	
Poor and below	89/467	0.99 (0.96, 1.02)	
Smoking status			0.08
Never	267/8531	1.01 (0.99, 1.03)	
Ever	194/3851	0.99 (0.97, 1.01)	
Alcohol drinking status			0.25
Never	326/8162	1.01 (0.99, 1.02)	
Ever	134/4233	0.99 (0.97, 1.02)	
BMI status			0.27
Underweight	52/593	1.02 (0.98, 1.07)	
Normal	237/6412	1.00 (0.99, 1.02)	
Overweight and obese	132/4749	0.99 (0.97, 1.01)	
Physical activity level			0.6
Low	179/3659	0.99 (0.97, 1.01)	
Medium	51/3903	0.98 (0.94, 1.02)	
High	84/3753	1.01 (0.97, 1.04)	
Missing	147/1138	1.02 (1.00, 1.04)	
History of comorbidities**			0.74
No	345/10782	1.00 (0.98, 1.01)	
Yes	115/1528	1.00 (0.97, 1.02)	

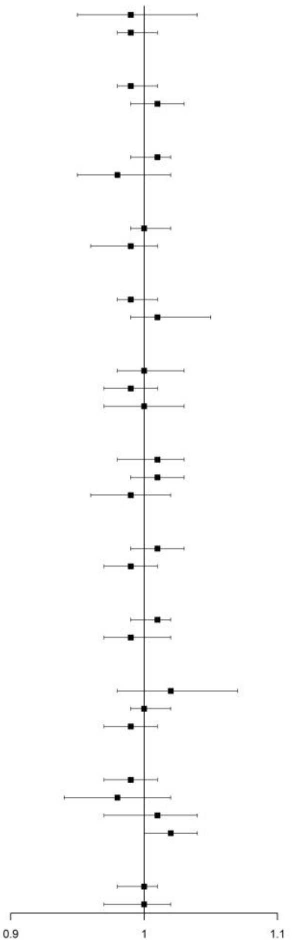

Figure S1. Forest plot of the stratified associations between continuous DQI-I score and risk of all-cause mortality by selected covariates

Abbreviations: BMI, body mass index; CI: confidence interval; DQI-I: Dietary Quality Index-International; HR: hazard ratio.

The multivariable-adjusted model adjusted for age, sex, baseline year, educational level, marriage status, household income, total energy intake, smoking status, drinking status, body mass index, physical activity level, and history of comorbidities.

*The cutoff point was set based on median age.

**Chronic diseases included hypertension, diabetes, myocardial infarction and stroke (cancer was not included due to majority of missing information).

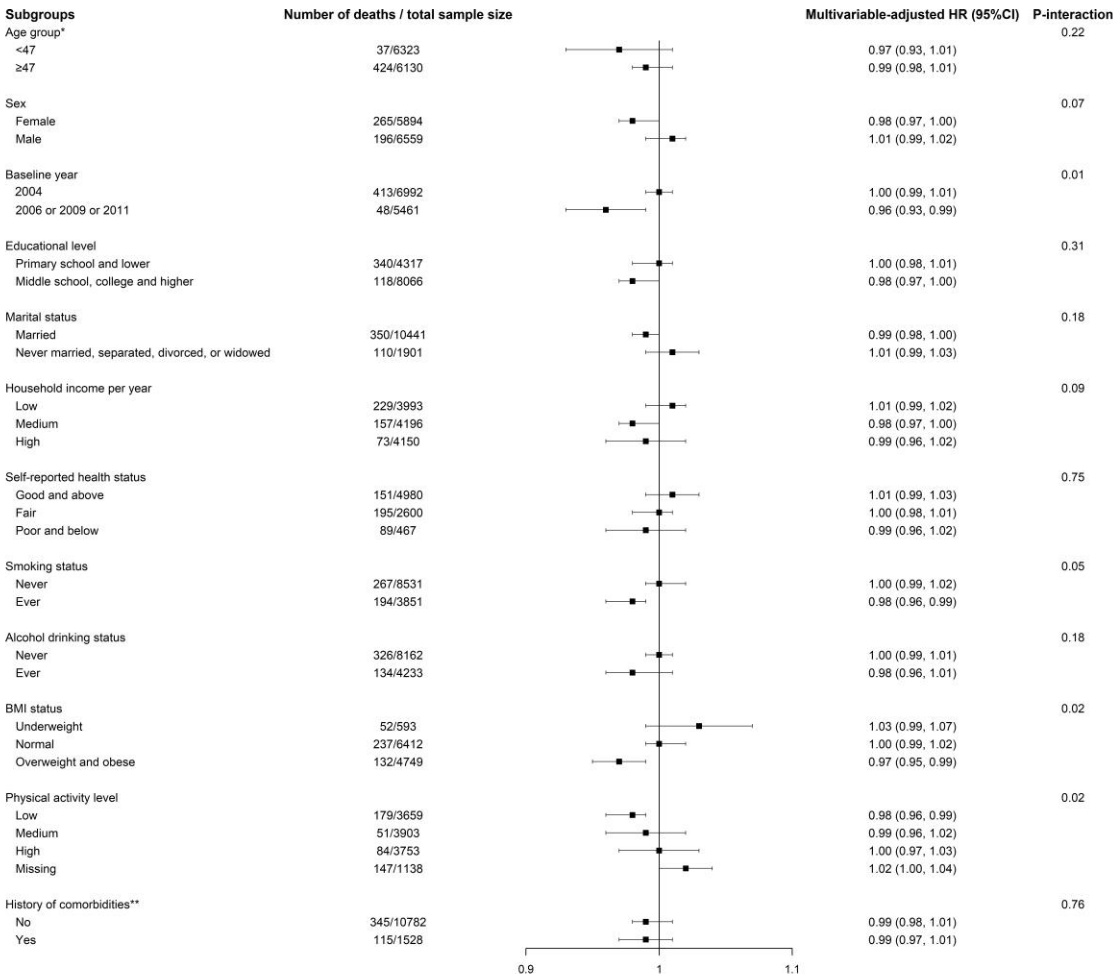

Figure S2. Forest plot of the stratified associations between continuous CHEI score and risk of all-cause mortality by selected covariates

Abbreviations: BMI, body mass index; CHEI, Chinese Health Eating Index; CI: confidence interval; HR: hazard ratio.

The multivariable-adjusted model adjusted for age, sex, baseline year, educational level, marriage status, household income, total energy intake, smoking status, drinking status, body mass index, physical activity level, and history of comorbidities.

*The cutoff point was set based on median age.

**Chronic diseases included hypertension, diabetes, myocardial infarction and stroke (cancer was not included due to majority of missing information).

Subgroups	Number of deaths / total sample size	Multivariable-adjusted HR (95%CI)	P-interaction
Age group*			0.15
<47	37/6323	1.14 (0.81, 1.59)	
≥47	424/6130	0.95 (0.87, 1.03)	
Sex			0.14
Female	265/5894	1.00 (0.90, 1.12)	
Male	196/6559	0.89 (0.78, 1.00)	
Baseline year			0.04
2004	413/6992	0.92 (0.85, 1.00)	
2006 or 2009 or 2011	48/5461	1.19 (0.93, 1.53)	
Educational level			0.09
Primary school and lower	340/4317	0.91 (0.83, 1.00)	
Middle school, college and higher	118/8066	1.05 (0.89, 1.23)	
Marital status			0.13
Married	350/10441	0.99 (0.90, 1.09)	
Never married, separated, divorced, or widowed	110/1901	0.84 (0.71, 0.99)	
Household income per year			0.49
Low	229/3993	0.91 (0.80, 1.03)	
Medium	157/4196	1.02 (0.89, 1.17)	
High	73/4150	0.96 (0.81, 1.15)	
Self-reported health status			0.15
Good and above	151/4980	0.85 (0.74, 0.98)	
Fair	195/2600	0.92 (0.81, 1.04)	
Poor and below	89/467	1.11 (0.91, 1.35)	
Smoking status			0.86
Never	267/8531	0.96 (0.86, 1.07)	
Ever	194/3851	0.96 (0.85, 1.08)	
Alcohol drinking status			0.25
Never	326/8162	0.92 (0.84, 1.02)	
Ever	134/4233	1.02 (0.87, 1.20)	
BMI status			0.01
Underweight	52/593	0.85 (0.64, 1.13)	
Normal	237/6412	0.88 (0.78, 0.98)	
Overweight and obese	132/4749	1.19 (1.01, 1.41)	
Physical activity level			0.91
Low	179/3659	0.94 (0.83, 1.07)	
Medium	51/3903	0.92 (0.71, 1.19)	
High	84/3753	0.99 (0.81, 1.22)	
Missing	147/1138	0.92 (0.79, 1.06)	
History of comorbidities**			0.19
No	345/10782	0.93 (0.84, 1.02)	
Yes	115/1528	1.06 (0.90, 1.25)	

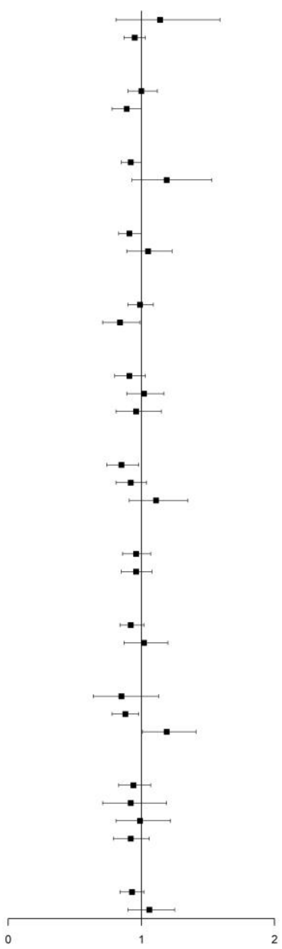

Figure S3. Forest plot of the stratified associations between continuous E-DII score and risk of all-cause mortality by selected covariates

Abbreviations: BMI, body mass index; CI: confidence interval; E-DII: energy adjusted-Dietary Inflammatory Index; HR: hazard ratio.

The multivariable-adjusted model adjusted for age, sex, baseline year, educational level, marriage status, household income, total energy intake, smoking status, drinking status, body mass index, physical activity level, and history of comorbidities.

*The cutoff point was set based on median age.

**Chronic diseases included hypertension, diabetes, myocardial infarction and stroke (cancer was not included due to majority of missing information).
