# Supplementary material for: Diet Quality and Mortality among Chinese Adults: Findings from the China Health and Nutrition Survey
Source: Nutrients. 2023 Dec 27;16(1):94. doi: 10.3390/nu16010094 (PMC10780502; doi:10.3390/nu16010094)
Supplement: Supplementary file 1 [file nutrients-16-00094-s001.zip › Supplemental tables.pdf]

Table S1. Components of Diet Quality Index-International (DQI-I) and scoring criteria

Component	Score	Scoring criteria
**Variety**	**0-20 points**	
Overall food group variety (meat/poultry/fish/eggs; dairy/beans; grain; fruit; vegetable)[a]	0-15 points	⩾1 serving per food group/d=15
		<1 serving consumption of 1 food group/d=12
		<1 serving consumption of 2 food groups/d=9
		<1 serving consumption of 3 food groups/d=6
		<1 serving consumption of 4 food groups/d=3
		<1 serving consumption of all these 5 food groups=0
Within-group variety for protein source (meat, poultry, fish, dairy, beans, eggs)[a]	0-5 points	⩾0.5 serving for no less than 3 different protein sources/d=5
		⩾0.5 serving for 2 different protein sources/d=3
		⩾0.5 serving for 1 protein source/d=1
		None=0
**Adequacy**[b]	**0-40 points**	
Vegetable group[a,c]	0-5 points	⩾3-5 servings/d=5, 0 serving/d=0
Fruit group[a,c]	0-5 points	⩾2-4 servings/d=5, 0 serving/d=0
Grain group[a,c]	0-5 points	⩾6-11 servings/d=5, 0 serving/d=0
Fiber[c]	0-5 points	⩾20-30 g/d=5, 0 g/d=0
Protein	0-5 points	⩾10% of energy/d=5, 0% of energy/d=0
Iron[d]	0-5 points	⩾100% AI/d=5, 0% AI/d=0
Calcium[d]	0-5 points	⩾100% AI/d=5, 0% AI/d=0
Vitamin C[e]	0-5 points	⩾100% RNI/d=5, 0% RNI/d=0
**Moderation**	**0-30 points**	
Total fat	0-6 points	⩽20% of total energy/d=6
		>20-30% of total energy/d=3
		>30% of total energy/d=0
Saturated fat	0-6 points	⩽7% of total energy/d=6
		>7-10% of total energy/d=3
		>10% of total energy/d=0
Cholesterol	0-6 points	⩽300 mg/d=6
		>300-400 mg/d=3

		>400 mg/d=0
Sodium	0-6 points	≤2400 mg/d=6
		>2400-3400 mg/d=3
		>3400 mg/d=0
Empty calorie foods[f]	0-6 points	≤3% of total energy/d=6
		>3-10% of total energy/d=3
		>10% of total energy/d=0
**Overall balance**	**0-10 points**	
Macronutrient ratio (carbohydrate:protein:fat)	0-6 points	55~65:10~15:15~25=6
		52~68:9~16:13~27=4
		50~70:8~17:12~30=2
		Otherwise=0
Fatty acid ratio (PUFA:MUFA:SFA)	0-4 points	P/S=1~1.5 and M/S=1~1.5=4
		Else if P/S =0.8~1.7 and M/S=0.8~1.7=2
		Otherwise=0

Abbreviations: AI, Adequate Intakes; PUFA, polyunsaturated fatty acids; RNI, Recommended Nutrient Intake; MUFA, monounsaturated fatty acids; SFA, saturated fatty acids; P/S, ratio of PUFA to SFA intake; M/S, ratio of MUFA to SFA intake.

[a]The Serving sizes for 5 different food groups originated from Dietary Guidelines for Americans 2005. Serving size for meat, poultry, fish: 3 oz meat, poultry or fish (85g). Serving size for dairy: 8 oz milk (226.8g); 1 cup yogurt (150g); 1.5 oz cheese (42.5g); 30g milk powder. Serving size for beans: 2 oz uncooked beans or tofu (56.7g); 4 oz cooked beans or tofu (113.4g). Serving size for egg: 1 egg (50g). Serving size for vegetable: 1 cup raw leafy vegetable (136g); 1/2 cup of other fresh or cooked vegetables (68g); 6 oz vegetable juice (170g). Serving size for fruit: 1 medium fruit (150g); 1/2 cup of fresh, frozen or canned fruit (68g); 1/4 cup of dried fruit (34g); 6 oz fruit juice (170g). Serving size for grains: 1 slice bread (25g); 1 oz dry cereal or rice (28.35g); 1/2 cup cooked rice or pasta or cereal (68g); 1 oz small cake (28.35g).

[b]Scores reflecting the consumption of food in this Adequacy dimension were distributed proportionally.

[c]The highest cutoff point varied from participants with different energy intake levels. The highest cutoff point for adequate vegetable intake was assigned to 3 servings/d for 0-2200kcal/d, 4 servings/d for 2200-2700kcal/d, and 5 servings/d for >2700kcal/d. The highest cutoff point for adequate fruit intake was assigned to 2 servings/d for 0-2200kcal/d, 3 servings/d for 2200-2700kcal/d, and 4 servings/d for >2700kcal/d. The highest cutoff point for adequate grains intake was assigned to 6 servings/d for 0-2200kcal/d, 9 servings/d for 2200-2700kcal/d, and 11 servings/d for >2700kcal/d. The highest cutoff point for adequate fiber intake was assigned to 20g/d for 0-2200kcal/d, 25g /d for 2200-2700 kcal/d, and 30g/d for >2700kcal/d.

[d]Scoring system based on the AI values for Chinese according to the Chinese Dietary Reference Intakes launched by the Chinese Nutrition Society in 2000.

[e]Scoring system based on the RNI values for Chinese according to the Chinese Dietary Reference Intakes launched by the Chinese Nutrition Society in 2000.

f. This section referred to cooking oil, alcohol, table sugar and candy.

Table S2. Standard portion size for food groups in the CHEI based on the Chinese Dietary Guidelines-2016

**Food categories[a]**	**Energy per SP (kcal)**	**Carbohydrate per SP (g)**	**Protein per SP (g)**	**Fat per SP (g)**
**Grains**	160-180	35-40	4-7	0-2
**Tubers**	80-90	18-22	0-4	0-1
**Vegetables**	15-35	3-7	1-3	0-1
**Fruits**	40-55	10-15	0-1	0-1
**Dairy**				
(whole fat % ⩾0.5%)	110	10-20	5-6	5-7
(low fat % <0.5%)	55	10-20	5-6	2-3
**Soybeans and products**	65-80	4-8	6-8	3-8
**Seeds and Nuts**	40-55	1-10	1-3	2-4
**Fish**	50-60	0-5	6-10	1-4
**Seafood**	35-50	0-5	6-10	1-4
**Meat and Poultry**				
(fat % <10%)	40-50	0-5	5-10	1-5
(fat % ⩾10%)	10-25	0-5	2-8	5-8
**Eggs**	40-50	1-3	7	5-10

Abbreviation: CHEI, Chinese Healthy Eating Index; SP, standard portion.

[a]The standard portion for grains was calculated by total energy of grain consumption divided by “energy per SP” (set as median value, e.g., grains, 170) for grains. Other food groups followed similar method of calculation while applying different references. For Tubers, the reference was median value of “carbohydrate per SP”. For vegetables, fruits, fish, seafood, meat and poultry, the reference was median value of “energy per SP”. For dairy, soybeans and products, eggs, the reference was median value of “protein per SP”. For seeds and nuts, the reference was median value of “fat per SP”.

Table S3. Chinese Healthy Eating Index food components and scoring criteria

Component	Scoring criteria[a]		
	0	5	10
**Adequacy**			
Total grains	0 →	≥2.5 SP/1000kcal	
Whole grains and mixed beans	0 →	≥0.6 SP/1000kcal	
Tubers	0 →	≥0.3 SP/1000kcal	
Total vegetables	0 →	≥1.9 SP/1000kcal	
Dark vegetables	0 →	≥0.9 SP/1000kcal	
Fruits	0 →	→	≥1.1 SP/1000kcal
Dairy	0 →	≥0.5 SP/1000kcal	
Soybeans	0 →	≥0.4 SP/1000kcal	
Fish and seafood	0 →	≥0.6 SP/1000kcal	
Poultry	0 →	≥0.3 SP/1000kcal	
Eggs	0 →	≥0.5 SP/1000kcal	
Seeds and nuts	0 →	≥0.4 SP/1000kcal	
**Limitation**			
Red meat	≥3.5 SP/1000kcal →	≤0.4 SP/1000kcal	
Cooking oils	≥32.6g/1000kcal →	→	≤15.6g/1000kcal
Sodium	≥3608mg/1000kcal →	→	≤1000mg/1000kcal
Added sugars[b]	≥20% of energy →	≤10% of energy	
Alcohol	≥25g (men)/15g (women) →	≤60g (men)/40g (women)	

Abbreviation: SP: standard portion.

[a]For fruits, cooking oils, sodium components, the maximum score was 10, while for the rest, the maximum score was 5. SPs were calculated based on the criteria in Supplemental Table 2. Scores reflecting the consumption between the highest and the lowest cutoff point were prorated linearly and the cutoffs were based on the updated Dietary Guidelines for Chinese (DGC-2016).

[b] Added sugars were included in cake, mooncake, soft drinks, sweetened juice, condensed milk, candy, and preserved fruits in our study

Table S4. Pearson correlation coefficients between DQI-I, CHEI, E-DII total score and component scores in the CHNS

	**DQI-I**	DQI-variety	DQI-adequacy	DQI-moderation	DQI-overall balance	**CHEI**	CHEI-adequacy	CHEI-limitation	**E-DII**
**DQI-I**	1	0.50**	0.74**	0.39**	0.34**	**0.53****	**0.31****	**0.49****	**-0.41****
DQI-variety	0.50**	1	0.42**	-0.40*	0.02*	0.39**	0.47**	0.01	-0.19**
DQI-adequacy	0.74**	0.42**	1	-0.11**	0.05**	0.49**	**0.45****	0.22**	-0.51**
DQI-moderation	0.39**	-0.40**	-0.11**	1	0.15**	0.03**	-0.29**	**0.49****	-0.02*
DQI-overall balance	0.34**	0.02*	0.05**	0.15**	1	0.15**	0.03**	0.23**	-0.05**
**CHEI**	**0.53****	0.39**	0.49**	0.03**	0.15**	1	0.84**	0.56**	**-0.43****
CHEI-adequacy	0.32**	0.47**	**0.45****	-0.29**	0.03**	0.84**	1	0.02*	-0.48**
CHEI-limitation	0.49**	0.01	0.22**	**0.49****	0.23**	0.56**	0.02*	11	-0.07**
**E-DII**	-0.41**	**-0.19****	**-0.51****	**-0.02***	**-0.05****	**-0.43****	**-0.48****	**-0.07****	1

Abbreviations: CHEI, Chinese Healthy Eating Index; DQI-I, Dietary Quality Index-International; E-DII: energy adjusted-Dietary Inflammatory Index.
*P<0.05; **P<0.01.

Table S5. Associations between DQI-I, CHEI, E-DII scores and all-cause mortality after removing deaths occurred in the first 3 years of follow-up (N=12,847)

	Quartile 1	Quartile 2	Quartile 3	Quartile 4	P_{trend}[a]	$HR_{continuous}$ (95%CI)[b]
**DQI-I**						
Total score, mean (range)	44.03 (24.18-49.10)	51.73 (49.11-54.07)	56.44 (54.08-58.89)	63.08 (58.90-82.82)		
Events/person-years	89/21129	110/22741	115/23716	80/22864		
HR (95%CI)[c]	Ref.	1.43 (1.07, 1.90)	1.51 (1.13, 2.02)	1.30 (0.94, 1.79)	0.22	1.01 (1.00, 1.02)
**Variety**						
Total score, mean (range)	6.90 (3.00-9.00)	10.10 (10.00-11.00)	12.77 (12.00-14.00)	16.68 (15.00-20.00)		
HR (95%CI)[c]	Ref.	1.12 (0.85, 1.46)	0.89 (0.67, 1.18)	0.72 (0.53, 0.99)	0.03	0.97 (0.94, 0.99)
**Adequacy**						
Total score, mean (range)	21.00 (9.43-23.93)	25.39 (23.94-27.08)	28.40 (27.09-29.81)	32.15 (29.81-40.00)		
HR (95%CI)[c]	Ref.	0.99 (0.75, 1.31)	1.06 (0.79, 1.41)	1.26 (0.92, 1.72)	0.09	1.02 (1.00, 1.05)
**Moderation**						
Total score, mean (range)	7.37 (0-9.00)	12.00 (12.00-12.00)	15.00 (15.00-15.00)	19.43 (18.00-27.00)		
HR (95%CI)[c]	Ref.	0.90 (0.65, 1.24)	1.04 (0.76, 1.43)	1.41 (1.06, 1.89)	<0.01	1.03 (1.01, 1.06)
**Overall balance**[d]						
Total score, mean (range)	1.02 (0-10.00)					
$HR_{>0\ vs.0}$ (95%CI)[c]	0.84 (0.67, 1.04)				0.06	0.94 (0.89, 1.00)
**CHEI**						
Total score, mean (range)	36.87 (17.23-42.13)	45.25 (42.12-48.21)	51.16 (48.22-54.47)	60.99 (54.48-88.51)		
Events/person-years	124/24141	128/23901	88/23060	54/19347		
HR (95%CI)[c]	Ref.	1.26 (0.98, 1.62)	1.06 (0.80, 1.40)	1.04 (0.74, 1.45)	0.77	1.00 (0.99, 1.01)
**Adequacy**						
Total score, mean (range)	13.45 (2.80-16.84)	19.08 (16.85-21.24)	23.91 (21.25-26.99)	33.34 (27.00-55.86)		
HR (95%CI)[c]	Ref.	1.01 (0.79, 1.29)	0.91 (0.69, 1.19)	0.69 (0.48, 0.99)	0.07	0.99 (0.97, 1.00)
**Limitation**						
Total score, mean (range)	18.92 (7.40-22.82)	24.89 (22.83-26.82)	28.56 (26.83-30.04)	32.14 (30.05-35.00)		
HR (95%CI)[c]	Ref.	1.32 (0.99, 1.75)	1.49 (1.11, 1.98)	1.44 (1.07, 1.94)	<0.01	1.03 (1.01, 1.05)
**E-DII**						
Mean (range)	–0.45 (–4.28-0.46)	0.91 (0.47-1.30)	1.66 (1.31-2.01)	2.50 (2.02-4.21)		
Events/person-years	89/20983	108/22521	100/23512	97/23434		
HR (95%CI)[c]	Ref.	1.08 (0.82, 1.44)	0.96 (0.72, 1.28)	0.84 (0.63, 1.13)	0.24	0.95 (0.87, 1.04)

Abbreviations: CHEI, Chinese Healthy Eating Index; CI: confidence interval; DQI-I, Dietary Quality Index-International; E-DII: energy adjusted-Dietary Inflammatory Index; HR, hazard ratio.
[a]The *P* value for trend were obtained from models with indices or component scores as continuous variables in the adjusted COX models.
[b]The hazard ratio indicated the change in the risk of death associated with 1-unit change in each dietary index and component score.
[c]Model was adjusted for age, sex, baseline year, educational level, marriage status, household income, total energy intake, smoking status, drinking status, body mass index, physical activity level, history of comorbidities which were same as those adjusted in Model 2.
[d]Since the majority got zero point for the "overall balance" component and could not be divided into quartiles, the hazard ratio was calculated by comparing participants scored above 0 to those scored 0.

Table S6. Associations between DQI-I, CHEI, E-DII scores and all-cause mortality in the complete case analysis (N=10,779)

	Quartile 1	Quartile 2	Quartile 3	Quartile 4	P_{trend}[a]	$HR_{continuous}$ (95%CI)[b]
**DQI-I**						
Total score, mean (range)	44.16 (25.19-49.18)	51.80 (49.19-54.17)	56.54 (54.18-58.98)	63.17 (58.99-82.00)		
Events/person-years	73/17620	78/19008	82/19873	56/19031		
HR (95%CI)[c]	Ref.	1.18 (0.85, 1.64)	1.17 (0.84, 1.62)	0.95 (0.66, 1.37)	0.40	0.99 (0.98, 1.01)
**Variety**						
Total score, mean (range)	6.93 (3.00-9.00)	10.10 (10.00-11.00)	12.79 (12.00-14.00)	16.71 (15.00-20.00)		
HR (95%CI)[c]	Ref.	1.15 (0.83, 1.57)	0.85 (0.61, 1.19)	0.77 (0.53, 1.11)	0.03	0.96 (0.93, 0.99)
**Adequacy**						
Total score, mean (range)	21.14 (10.39-24.09)	25.72 (24.10-27.18)	28.50 (27.19-29.88)	32.21 (29.89-40.00)		
HR (95%CI)[c]	Ref.	0.95 (0.69, 1.32)	0.97 (0.70, 1.36)	1.16 (0.82, 1.66)	0.85	1.00 (0.97, 1.03)
**Moderation**						
Total score, mean (range)	11.51 (6.00-12.00)	15.00 (15.00-15.00)	19.42 (18.00-21.00)	25.24 (24.00-30.00)		
HR (95%CI)[c]	Ref.	0.94 (0.55, 1.60)	0.88 (0.57, 1.36)	1.18 (0.75, 1.84)	0.20	1.02 (0.99, 1.05)
**Overall balance**[d]						
Total score, mean (range)	1.00 (0-10.00)					
$HR_{>0 vs 0}$ (95%CI)[c]	0.65 (0.50, 0.84)				<0.01	0.89 (0.82, 0.96)
**CHEI**						
Total score, mean (range)	37.06 (17.23-42.41)	45.53 (42.42-48.46)	51.54 (48.47-54.90)	61.42 (54.91-88.51)		
Events/person-years	98/20162	95/20012	58/19293	38/16065		
HR (95%CI)[c]	Ref.	1.12 (0.84, 1.49)	0.78 (0.56, 1.09)	0.84 (0.57, 1.23)	0.08	0.99 (0.97, 1.00)
**Adequacy**						
Total score, mean (range)	13.61 (2.80-17.08)	19.35 (17.09-21.63)	24.30 (21.64-27.41)	33.76 (27.42-55.86)		
HR (95%CI)[c]	Ref.	0.89 (0.67, 1.18)	0.79 (0.57, 1.09)	0.47 (0.29, 0.74)	<0.01	0.97 (0.95, 0.99)
**Limitation**						
Total score, mean (range)	18.92 (7.40-22.84)	24.91 (22.85-26.85)	28.57 (26.86-30.02)	32.12 (30.03-35.00)		
HR (95%CI)[c]	Ref.	1.33 (0.96, 1.85)	1.22 (0.86, 1.72)	1.30 (0.92, 1.84)	0.14	1.02 (0.99, 1.04)
**E-DII**						
Mean (range)	–0.48 (–4.28-0.45)	0.90 (0.46-1.29)	1.65 (1.30-2.00)	2.49 (2.01-4.21)		
Events/person-years	72/17420	71/18903	72/19657	74/19551		
HR (95%CI)[c]	Ref.	0.92 (0.66, 1.28)	0.85 (0.61, 1.18)	0.82 (0.59, 1.14)	0.61	0.97 (0.88, 1.08)

Abbreviations: CHEI, Chinese Healthy Eating Index; CI: confidence interval; DQI-I, Dietary Quality Index-International; E-DII: energy adjusted-Dietary Inflammatory Index; HR, hazard ratio.

[a]The P value for trend was obtained from models with indices or component scores as continuous variables in the adjusted COX model.
[b]The hazard ratio indicated the change in the risk of death associated with 1-unit change in each index and component score.
[c]The adjusted model was adjusted for age, sex, baseline year, educational level, marriage status, household income, total energy intake, smoking status, drinking status, body mass index, physical activity level, history of comorbidities.
[d]Since the majority got zero point for the "overall balance" component and could not be divided into quartiles, the hazard ratio was calculated by comparing participants scored greater than 0 to those scored 0.

Table S7. Associations between DQI-I, CHEI, E-DII scores and all-cause mortality without adjustment of BMI status (N=12,914)

	Quartile 1	Quartile 2	Quartile 3	Quartile 4	P_{trend}[a]	$HR_{continuous}$ (95%CI)[b]
**DQI-I**						
Total score, mean (range)	44.01 (24.18-49.09)	51.72 (49.10-54.06)	56.42 (54.07-58.87)	63.06 (58.88-82.82)		
HR (95%CI)[c]	Ref.	1.26 (0.97, 1.63)	1.29 (0.99, 1.67)	1.03 (0.77, 1.39)	0.97	1.00 (0.99, 1.01)
**Variety**						
HR (95%CI)[c]	Ref.	1.02 (0.79, 1.31)	0.86 (0.67, 1.12)	0.67 (0.50, 0.89)	<0.01	0.96 (0.93, 0.99)
**Adequacy**						
HR (95%CI)[c]	Ref.	0.92 (0.71, 1.18)	0.96 (0.73, 1.24)	1.06 (0.80, 1.41)	0.64	1.01 (0.98, 1.03)
**Moderation**						
HR (95%CI)[c]	Ref.	0.93 (0.69, 1.26)	1.01 (0.75, 1.35)	1.40 (1.07, 1.83)	<0.01	1.03 (1.01, 1.05)
**Overall balance**[d]						
$HR_{>0 \text{ vs. } 0}$ (95%CI)[c]	0.80 (0.65, 0.98)				0.02	0.93 (0.88, 0.99)
**CHEI**						
Total score, mean (range)	36.82 (17.23-42.12)	45.22 (42.13-48.18)	51.14 (48.19-54.45)	60.97 (54.46-88.51)		
HR (95% CI)[c]	Ref.	1.16 (0.92, 1.46)	0.97 (0.75, 1.25)	0.88 (0.65, 1.21)	0.25	0.99 (0.98, 1.01)
**Adequacy**						
HR (95% CI)[c]	Ref.	0.93 (0.74, 1.17)	0.85 (0.66, 1.09)	0.57 (0.41, 0.81)	<0.01	0.97 (0.96, 0.99)
**Limitation**						
HR (95% CI)[c]	Ref.	1.25 (0.97, 1.62)	1.37 (1.05, 1.77)	1.32 (1.00, 1.73)	0.02	1.02 (1.00, 1.04)
**E-DII**						
Mean (range)	–0.45 (–4.28-0.47)	0.91 (0.48-1.31)	1.66 (1.32-2.02)	2.50 (2.03-4.21)		
HR (95% CI)[c]	Ref.	1.02 (0.79, 1.32)	0.88 (0.67, 1.15)	0.85 (0.65, 1.10)	0.20	0.95 (0.87, 1.03)

Abbreviations: CHEI, Chinese Healthy Eating Index; CI, confidence interval; DQI-I, Dietary Quality Index-International. E-DII: energy adjusted-Dietary Inflammatory Index; HR, hazard ratio.
[a]The *P* value for trend was obtained from models with indices or component scores as continuous variables in the adjusted COX models.
[b]The hazard ratio indicated the change in the risk of death associated with 1-unit change in each index and component score.
[c]The model was adjusted for age, sex, baseline year, educational level, marriage status, household income, total energy intake, smoking status, drinking status, physical activity level, history of comorbidities.

[d]Since the majority got zero point for the "overall balance" component and could not be divided into quartiles, the hazard ratio was calculated by comparing participants scored greater than 0 to those scored 0.

Table S8. Associations between DQI-I, CHEI, E-DII scores and all-cause mortality among participants without history of comorbidities at baseline (N=11,246)

	Quartile 1	Quartile 2	Quartile 3	Quartile 4	P_{trend}[a]	$HR_{continuous}$ (95%CI)[b]
**DQI-I**						
Total score, mean (range)	44.21 (24.18-49.25)	51.87 (49.26-54.19)	56.51 (54.20-58.94)	63.05 (58.95-82.00)		
Events/person-years	88/18676	89/20352	99/21175	70/20355		
HR (95%CI)[c]	Ref.	1.11 (0.82, 1.51)	1.26 (0.93, 1.71)	1.11 (0.79, 1.55)	0.88	1.00 (0.98, 1.01)
**Variety**						
Total score, mean (range)	6.89 (3.00-9.00)	10.09 (10.00-11.00)	12.76 (12.00-14.00)	16.63 (15.00-20.00)		
HR (95%CI)[c]	Ref.	1.05 (0.79, 1.40)	0.99 (0.73, 1.33)	0.73 (0.52, 1.03)	0.05	0.97 (0.94, 1.00)
**Adequacy**						
Total score, mean (range)	21.02 (9.43-23.94)	25.59 (23.95-27.06)	28.37 (27.07-29.74)	32.02 (29.75-40.00)		
HR (95%CI)[c]	Ref.	0.93 (0.69, 1.26)	0.93 (0.68, 1.27)	1.34 (0.97, 1.85)	0.28	1.02 (0.99, 1.05)
**Moderation**						
Total score, mean (range)	7.40 (0-9.00)	12.00 (12.00-12.00)	15.00 (15.00-15.00)	19.45 (18.00-27.00)		
HR (95%CI)[c]	Ref.	0.85 (0.60, 1.22)	0.99 (0.71, 1.38)	1.15 (0.84, 1.57)	0.22	1.02 (0.99, 1.04)
**Overall balance**[d]						
Total score, mean (range)	1.04 (0-10.00)					
$HR_{>0\ vs.\ 0}$ (95%CI)[c]	0.78 (0.62, 0.99)				0.02	0.92 (0.86, 0.99)
**CHEI**						
Total score, mean (range)	36.76 (18.30-42.03)	45.12 (42.04-48.01)	50.92 (48.02-54.20)	60.44 (54.21-88.51)		
Events/person-years	116/21450	114/21107	74/20607	42/17394		
HR (95%CI)[c]	Ref.	1.21 (0.93, 1.58)	0.91 (0.68, 1.23)	0.90 (0.62, 1.30)	0.25	0.99 (0.98, 1.01)
**Adequacy**						
Total score, mean (range)	13.23 (3.13-16.52)	18.78 (16.53-20.89)	23.46 (20.90-26.41)	32.62 (26.42-53.51)		
HR (95%CI)[c]	Ref.	0.99 (0.76, 1.28)	0.80 (0.59, 1.07)	0.73 (0.50, 1.07)	0.01	0.98 (0.96, 0.99)
**Limitation**						
Total score, mean (range)	19.14 (10.00-23.05)	25.09 (23.06-27.02)	28.71 (27.03-30.16)	32.22 (30.17-35.00)		
HR (95%CI)[c]	Ref.	1.14 (0.85, 1.54)	1.39 (1.03, 1.87)	1.16 (0.84, 1.59)	0.19	1.02 (0.99, 1.04)
**E-DII**						

Mean (range)	–0.39 (–4.05-0.51)	0.95 (0.52-1.34)	1.69 (1.35-2.03)	2.51 (2.04-4.21)		
Events/person-years	87/18919	95/20083	80/20877	84/20680		
HR (95%CI)[c]	Ref.	1.01 (0.75, 1.35)	0.82 (0.60, 1.11)	0.80 (0.59, 1.09)	0.09	0.92 (0.83, 1.01)

Abbreviations: CHEI, Chinese Healthy Eating Index; CI, confidence interval; DQI-I, Dietary Quality Index-International; E-DII: energy adjusted-Dietary Inflammatory Index; HR, hazard ratio.

[a]The *P* value for trend was obtained from models with indices or component scores as continuous variables in the adjusted COX model.

[b]The hazard ratio indicated the change in the risk of death associated with 1-unit change in each index and component score.

[c]Model was adjusted for age, sex, baseline year, educational level, marriage status, household income, total energy intake, smoking status, drinking status, body mass index, physical activity level.

[d]Since the majority got zero point for the "overall balance" component and could not be divided into quartiles, the hazard ratio was calculated by comparing participants scored greater than 0 to those scored 0.

Table S9. Associations between DQI-I and all-cause mortality among subjects with daily average energy intake greater than 1700kcal[a] (N=8,886)

	Quartile 1	Quartile 2	Quartile 3	Quartile 4	P_{trend}[b]	$HR_{continuous}$ (95%CI)[c]
**DQI-I**						
Total score, mean (range)	46.22 (27.10-50.60)	53.03 (50.61-55.28)	57.43 (55.29-59.72)	63.81 (59.73-82.82)		
Events/person-years	77/16110	89/16780	84/17230	56/16220		
HR (95%CI)[d]	Ref.	1.08 (0.80, 1.47)	1.19 (0.87, 1.63)	0.85 (0.60, 1.20)	0.24	0.99 (0.97, 1.01)
**Variety**						
Total score, mean (range)	7.05 (3.00-9.00)	11.00 (10.00-12.00)	14.47 (13.00-15.00)	17.59 (17.00-20.00)		
HR (95%CI)[d]	Ref.	1.08 (0.80, 1.46)	0.91 (0.63, 1.30)	0.70 (0.46, 1.06)	0.02	0.96 (0.93, 0.99)
**Adequacy**						
Total score, mean (range)	22.73 (10.39-25.26)	26.70 (25.27-27.98)	29.15 (27.99-30.35)	32.63 (30.36-40.00)		
HR (95%CI)[d]	Ref.	0.98 (0.72, 1.33)	1.06 (0.77, 1.45)	1.10 (0.79, 1.54)	0.87	1.00 (0.97, 1.04)
**Moderation**						
Total score, mean (range)	5.54 (0-6.00)	9.00 (9.00-9.00)	13.46 (12.00-15.00)	19.36 (18.00-27.00)		
HR (95%CI)[d]	Ref.	1.11 (0.69, 1.81)	1.00 (0.67, 1.49)	1.22 (0.81, 1.84)	0.48	1.01 (0.98, 1.04)
**Overall balance**[e]						
Total score, mean (range)	1.07 (0-10.00)					
HR (95%CI)[d]	0.79 (0.61, 1.01)				0.05	0.93 (0.86, 1.00)

Abbreviations: CI, confidence interval; DQI-I, Dietary Quality Index-International. HR, hazard ratio.

[a]The lowest cutoff points for vegetable group, fruit group, grain group and fiber were set based on an average energy intake of no less than 1700kcal/d in DQI-I in **Supplemental Table 1**. The sensitivity analysis removed participants who did not meet the criteria.

[b]The *P* value for trend were obtained from models with indices or component scores as continuous variables in the adjusted COX model.

[c]The hazard ratio indicated the change in the risk of death associated with 1-unit change in each index and component score.

[d]Model 2 adjusted for age, sex, baseline year, educational level, marriage status, household income, total energy intake, smoking status, drinking status, body mass index, physical activity level, history of comorbidities.

[e]Since the majority got zero point for the "overall balance" component and could not be divided into quartiles, the hazard ratio was calculated by comparing participants scored greater than 0 to those scored 0.

Table S10. Associations between CHEI with standard portion calculated based on total energy and all-cause mortality[a] (N=12,914)

	Quartile 1	Quartile 2	Quartile 3	Quartile 4	P_{trend}[b]	$HR_{continuous}$ (95%CI)[c]
**CHEI**						
Total score, mean (range)	36.82 (17.23-42.12)	45.22 (42.13-48.18)	51.14 (48.19-54.45)	60.97 (54.46-88.51)		
Events/person-years	154/24223	147/23920	101/23155	59/19303		
HR (95%CI)[d]	Ref.	1.18 (0.94, 1.49)	0.99 (0.76, 1.28)	0.92 (0.67, 1.26)	0.28	0.99 (0.98, 1.01)
**Adequacy**						
Total score, mean (range)	13.57 (2.88-17.02)	19.28 (17.03-21.53)	24.21 (21.53-27.36)	33.85 (27.37-56.94)		
HR (95%CI)[d]	Ref.	1.00 (0.80, 1.25)	0.85 (0.66,1.09)	0.60 (0.43, 0.85)	<0.01	0.98 (0.96, 0.99)
**Limitation**						
Total score, mean (range)	18.91 (7.40-22.80)	24.88 (22.81-26.81)	28.55 (26.82-30.03)	32.13 (30.04-35.00)		
HR (95%CI)[d]	Ref.	1.25 (0.96, 1.62)	1.34 (1.04, 1.75)	1.29 (0.98, 1.70)	0.03	1.02 (1.00, 1.04)

Abbreviations: CHEI, Chinese Healthy Eating Index; CI, confidence interval; HR, hazard ratio.

[a]The sensitivity analysis calculated standard portion by harmonizing consumption of all food groups with "energy per SP": Standard portion=total energy intake of each food group divided by median value of "energy per SP" of each food group.

[b]The *P* value for trend were obtained from models with indices or component scores as continuous variables in the adjusted COX model.

[c]The hazard ratio indicated the change in the risk of death associated with 1-unit change in each index and component score.

[d]Model was adjusted for age, sex, baseline year, educational level, marriage status, household income, total energy intake, smoking status, drinking status, body mass index, physical activity level, history of comorbidities.
